# Risk factors and outcome in dogs with recurrent massive hepatocellular carcinoma: A Veterinary Society of Surgical Oncology case–control study

**DOI:** 10.1111/vco.12824

**Published:** 2022-05-15

**Authors:** Janis M. Lapsley, Vincent Wavreille, Sabrina Barry, Josephine A. Dornbusch, Carolyn Chen, Haley Leeper, Judith Bertran, Diane Scavelli, Julius M. Liptak, Chris Wood, Shelly Shamir, Claire Rosenbaum, Vincenzo Montinaro, Brandan Wustefeld‐Janssens, Allyson Sterman, Colin Chik, Ameet Singh, Josh Collins, Laura E. Selmic

**Affiliations:** ^1^ Department of Veterinary Clinical Sciences, College of Veterinary Medicine The Ohio State University Columbus Ohio USA; ^2^ VetSpecialistes SA Grand‐Saconnex Switzerland; ^3^ Virginia‐Maryland College of Veterinary Medicine Virginia Tech Blacksburg Virginia USA; ^4^ Department of Small Animal Medicine and Surgery, College of Veterinary Medicine University of Georgia Athens Georgia USA; ^5^ Department of Clinical Sciences, Carlson College of Veterinary Medicine Oregon State University Corvallis Oregon USA; ^6^ Department of Small Animal Clinical Sciences, College of Veterinary Medicine The University of Florida Gainesville Florida USA; ^7^ Department of Clinical Sciences and Advanced Medicine, Matthew J. Ryan Veterinary Hospital University of Pennsylvania Philadelphia Pennsylvania USA; ^8^ Flint Animal Cancer Center and Department of Clinical Sciences, College of Veterinary Medicine and Biomedical Sciences Colorado State University Fort Collins Colorado USA; ^9^ Capital City Small Animal Mobile Surgery Ottawa Ontario Canada; ^10^ Alta Vista Animal Hospital Ontario Canada; ^11^ Department of Veterinary Medicine and Surgery, College of Veterinary Medicine University of Minnesota Twin Cities Minnesota USA; ^12^ Soft Tissue Surgery Department Clinica Veterinaria Malpensa‐AniCura Samarate Italy; ^13^ Department of Small Animal Clinical Sciences, College of Veterinary Medicine and Biomedical Sciences Texas A&M University Texas USA; ^14^ Department of Veterinary Clinical Sciences Purdue University College of Veterinary Medicine West Lafayette Indiana USA; ^15^ College of Veterinary Medicine Cornell University Ithaca New York USA; ^16^ Department of Clinical Studies, Ontario Veterinary College University of Guelph Guelph Ontario Canada; ^17^ Surgery Department Metropolitan Veterinary Hospital Copley Ohio USA

**Keywords:** dog, hepatocellular carcinoma, local recurrence, survival

## Abstract

Local recurrence after surgical excision of canine massive hepatocellular carcinoma (HCC) has been poorly studied in veterinary medicine with scant information published regarding risk factors for and outcome following recurrence. The aim of this case–control study was to describe the time to recurrence, evaluate potential risk factors for recurrence, and report the outcome in dogs with massive HCC. Medical records for 75 dogs who developed recurrence and 113 dogs who did not develop recurrence were reviewed. Statistical analyses were performed to determine risk factors for recurrence as well as the median time to develop recurrence and overall survival time (OS). None of the risk factors evaluated were significant for the development of recurrence. The median time to develop recurrence was 367 days (range 32–2096 days). There was no significant difference in median OS for dogs who developed recurrence vs. those who did not (851 vs. 970 days). For dogs with recurrent HCC, treatment at recurrence trended toward prolonged OS but was not significantly different from dogs not undergoing treatment at recurrence. There was no significant difference in median OS for dogs with histologically complete vs. incomplete tumour excision (990 vs. 903 days). Although specific risk factors for recurrence were not identified, elevations in liver values were noted in patients with recurrent disease and could act as a noninvasive surveillance tool. Recurrence was noted earlier in dogs who had routine post‐operative surveillance (228 vs. 367 days). Routine surveillance for recurrence is recommended especially in dogs where further intervention is possible and should extend beyond 1 year. Patients with massive HCC have a good long‐term prognosis regardless of incomplete excision, pulmonary metastasis, or recurrent local disease.

## 1 INTRODUCTION

Primary liver tumours are rare in dogs, representing less than 1.5% of all canine neoplasia.^1^, ^2^ Malignant tumours of the liver include hepatocellular carcinoma (HCC), cholangiocellular carcinoma, neuroendocrine tumours, and sarcomas. Hepatocellular carcinoma is the most common, accounting for approximately 50%–77% of primary hepatobiliary tumours in dogs.^2^, ^3^ Hepatocellular carcinoma is an epithelial tumour primarily arising from hepatocytes and is usually found in older dogs. No sex predisposition or specific risk factors have been recognized, although male dogs may be overrepresented.^4^ Retrospective studies have identified an increased risk of HCC development in Welsh Corgis and beagles, Scottish Terriers with vacuolar hepatopathy, and dogs with hyperadrenocorticism.^5^, ^6^

Three morphologic subtypes of HCC are described: massive, nodular, and diffuse. Nodular and diffuse morphologies present as multifocal or diffuse infiltrating tumours and historically carry a poorer prognosis.^2^ The massive morphology is defined as a large tumour affecting a single liver lobe and represents 53%–83% of all canine HCC.^2^ Massive HCC is most often reported in the left division, is typically slow‐growing, and reported to have a favourable prognosis with prolonged survival times following surgical excision. Survival greater than 1400 days irrespective of surgical margins and 765 days with incomplete margins is reported.^1^, ^7^ Factors reported to be associated with a poorer prognosis include lack of surgical treatment, right‐sided location, or disease in the quadrate lobe.^8^ Similarly, elevated serum alanine aminotransferase (ALT), aspartate aminotransferase (AST), alkaline phosphatase (ALP) to AST ratio, blood urea nitrogen (BUN), potassium, and gamma‐glutamyl transferase (GGT) have been identified as potential poor prognostic indicators.^1^, ^8^ The distant metastatic rate for massive HCC is variable ranging between 0% and 37%.^2^

Information regarding local recurrence rates following complete or incomplete resection, risk factors for local recurrence, and outcome following treatment of recurrent lesions are not well described. One study reported progressive local disease in 3 of 25 dogs with complete excision and 7 of 12 dogs with incomplete excision and included dogs whose recurrence was multifocal.^7^ Another study reported no local recurrence in 42 dogs with 4 having undergone incomplete resection.^1^ A study evaluating long term outcomes in dogs undergoing liver lobectomy for any liver tumour found the most common cause of tumour related death to be recurrence which occurred in 14 of 19 dogs that died.^9^ No study reported time to recurrence, potential risk factors for recurrence, survival following recurrence, or any further treatment of recurrent cases. The objective of this retrospective case–control study was to describe the time to local recurrence, evaluate the potential risk factors for local recurrence, and report the outcome in dogs with massive HCC in a large cohort of dogs.

## 2 METHODS

After study design approval by the Veterinary Society of Surgical Oncology (VSSO) research committee, case submissions were solicited from the VSSO listserv. Contributors were asked to search for cases and controls which met the inclusion criteria. For inclusion as a case, dogs needed to be diagnosed with locally recurrent HCC confirmed via cytologic, histopathologic, or imaging diagnoses, and have previously undergone surgical excision of a single massive HCC lesion between 1 January 2004 and 31 December 2018. A group of control cases were also collected. For inclusion as a control, dogs needed to have undergone surgical excision of a single massive HCC lesion without documented recurrence over the subsequent 6 months or greater. Cases or controls were excluded if multiple separate liver masses were found at initial surgery, or for cases, if recurrence was diffuse or spanned more than two adjacent liver divisions (right, central, or left). Cases or controls without complete medical records or where appropriate follow‐up was unavailable were also excluded. Medical records for cases and controls that met the inclusion criteria were reviewed by each institutional contributor. Data collected from medical records of cases and controls included: signalment, body weight, diagnostics from initial presentation (bloodwork, thoracic and abdominal imaging, fine needle aspirate [FNA] cytology of the liver mass), surgical information from resection of the primary massive HCC lesion (largest tumour dimensions, tumour location, intraoperative and postoperative complications encountered), and histopathology following primary surgery (margins, degree of overall differentiation, nuclear pleomorphism, amount of necrosis, mitotic count). For cases, the date of recurrence and reason for reevaluation leading to recognition of recurrence, workup performed at the time of recurrence (bloodwork, imaging, FNA cytology), and type of management for recurrence (surgery vs. medical) was collected. Local recurrence was defined as the presence of a mass in the same liver lobe (when the lobe was not completely resected at initial surgery), an immediately adjacent lobe, or the same or immediately adjacent liver division as the initial mass. If surgical treatment was performed, the location of recurrence, intraoperative and postoperative complications and histopathology findings of the recurrent mass were recorded. When available, the date of last follow‐up and date of death were recorded with patient outcome reported as alive, dead, or lost to follow up. Cause of death most likely related to HCC recurrence and necropsy findings were recorded when available.

Fine needle aspirate cytology reports provided by each institution were evaluated by a single author (JML) and categorized according to the following: carcinoma, hepatocellular carcinoma, or hepatocellular neoplasia; marked atypia, likely consistent with carcinoma or hepatocellular neoplasia; mild atypia, vacuolar hepatopathy, hepatopathy, or hyperplasia; and inconclusive or nondiagnostic, including necrosis and well differentiated hepatocytes.

Histopathology reports were evaluated by a single author (JML) to extract information regarding completeness of surgical margins, the overall degree of differentiation, nuclear pleomorphism, amount of necrosis, and mitotic count. The mitotic count was reported as the number of mitosis per 10 high‐powered fields. Surgical margins were reported as histologically incomplete (neoplastic cells present at the margin) or complete (no neoplastic cells present at the margin) and distance to closest surgical margin was reported in millimetres when available. Due to high variability in reporting of the degree of differentiation, nuclear pleomorphism, and amount of necrosis from each institution the following categorizations were created. The degree of differentiation was categorized as well if the terms ‘well differentiated, low grade, organized, encapsulated and/or well demarcated’ were listed in the histopathology report. Differentiation was categorized as moderate if the terms ‘moderately differentiated, intermediate grade, moderately encapsulated and/or moderately demarcated’ were listed in the histopathology report. Differentiation was categorized as poor if the terms ‘poorly differentiated, high grade, disorganized, irregular, or unencapsulated’ were listed in the histopathology report. Nuclear pleomorphism was categorized as mild if the histopathology report stated rare to no multinucleation, 1–2 nucleoli, well defined cell border, mild anisokaryosis, and/or mild anisocytosis. Nuclear pleomorphism was categorized as moderate if the histopathology report stated moderate multinucleation, 2–3 nucleoli, moderate anisokaryosis, and/or moderate anisocytosis. Nuclear pleomorphism was categorized as marked if the histopathology report stated frequent bi‐, tri‐, or multinucleation, marked anisokaryosis, and/or marked anisocytosis. The amount of necrosis was categorized as mild if the terms ‘occasional, foci, scattered, some, or <25%’ were listed in the histopathology report. The amount of necrosis was categorized as moderate if the terms ‘regional, multiple focal regions, or <50%’ were listed in the histopathology report. The amount of necrosis was categorized as marked if the terms ‘expansile, extensive, large, or >50%’ were listed in the histopathology report.

Surgical complications were reported by each institution and categorized by a single author (JML) using the Classification of Intraoperative Complications (CLASSIC) as described in Table 1.^10^ As the inclusion criteria required a minimum of 6 months follow‐up, controls experiencing death short‐term (grade 4 intra‐ or post‐operative complications) were excluded. Intraoperative complications included those sustained during anaesthesia and surgery. Postoperative complications occurred after recovery from anaesthesia and within 2 weeks of surgery.

**TABLE 1:** Classification of intraoperative complications (CLASSIC) criteria

Grade	Complication
0	No complication, no deviation from the ideal operative course
1	Any deviation from the ideal operative course; without need for additional treatment or intervention
2	Any deviation from the ideal operative course; need for minor additional treatment or intervention, not life threatening and not leading to permanent disability
3	Any deviation from the ideal operative course; need for any additional treatment or intervention, life threatening and/or leading to permanent disability
4	Any deviation from the ideal operative course; death of the patient

*Source*: Adapted from Reference [10].

The time from initial surgery to local recurrence was calculated as the number of days from the date of initial surgery to the date of detection of local recurrence. Timing of follow‐up diagnostics was not standardized and was performed at the discretion of the overseeing clinician either due to clinical or laboratory abnormalities or according to individual institution surveillance protocols. Routine surveillance protocols varied by institution and ranged from no recommended monitoring to abdominal imaging every 3 to 6 months. The overall survival time (OS) for recurrent and control cases was calculated as the number of days from the date of first surgery to the date of death (all causes), or last follow‐up if alive. The endpoint for OS calculations was defined as death or euthanasia resulting from any cause. Kaplan–Meier survival analyses were performed using commercially available statistical software (GraphPad Prism 8.0.0, GraphPad Software, San Diego, California USA) to determine median OS and survival rates at 1, 2, 3, and 5 years for cases and controls. Log‐rank test was used to compare survival distribution between control and recurrent cases; recurrent cases who underwent surgery at the time of local recurrence and those who did not; recurrent cases with suspected pulmonary metastasis at the time of recurrence and those without; and complete versus incomplete resection. Dogs that were alive at last follow‐up or lost to follow‐up were censored from survival analysis.

For purposes of risk factor analysis for local recurrence, one case was randomly selected for every two controls submitted from the same institution using a commercially available randomization tool (Microsoft Corporation, Microsoft Excel 2018). Univariable conditional logistic regression was performed using commercially available statistical software (Excel). Risk factors evaluated for recurrence included sex, breed, age, body weight, tumour size, tumour location, FNA cytology findings, histopathologic findings, mitotic count, and completeness of excision. Risk factors for the occurrence of surgical complications were evaluated and included liver division, size of the mass, and patient body weight.

*Cell validation statement*: No cell lines were used in this study.

## 3 RESULTS

Cases and controls were collected from multiple institutions, including private practice (3) and academic veterinary centres (9) in the United States (9), Canada (2), and Europe (1). A total of 92 recurrent cases were submitted. Of these, 17 cases were excluded due to incomplete medical records (7), the presence of multiple liver lobe involvement at initial surgery (9), or the presence of rapid diffuse recurrence (1) resulting in a total of 75 recurrent cases. A total of 163 control cases were submitted. Fifty controls were excluded due to incomplete medical records (7), presence of multiple liver lobe involvement at initial surgery (9), or follow‐up period of less than 6 months (34) resulting in 113 control cases. One case was randomly selected for every two controls submitted by each institution, resulting in 43 recurrent cases and 86 controls evaluated for local recurrence risk factor analysis. Demographics at the time of the initial surgery are summarized in Table 2.

**TABLE 2:** Demographics at initial surgery in 75 dogs experiencing HCC tumour recurrence and 113 dogs not experiencing recurrence.

	Cases (*n* = 75)	Controls (*n* = 113)
Contributing institution		
Virginia Maryland College of Veterinary Medicine	11 (14.7%)	24 (21.2%)
University of Minnesota	15 (20.0%)	0 (0%)
University of Florida	9 (12.0%)	7 (6.2%)
Texas A&M University	7 (9.3%)	6 (5.3%)
Oregon State University	7 (9.3%)	11 (9.7%)
University of Pennsylvania	6 (8.0%)	11 (9.7%)
The Ohio State University	6 (8.0%)	17 (15%)
Alta Vista Animal Hospital	5 (6.7%)	11 (9.7%)
Ontario Veterinary College	4 (5.3%)	2 (1.8%)
Cornell University	4 (5.3%)	9 (8.0%)
Metropolitan Veterinary Hospital	1 (1.3%)	0 (0%)
Clinica Veterinaria Malpensa‐AniCura	0 (0%)	15 (13.3%)
Sex		
Intact female	1 (1.3%)	1 (0.9%)
Spayed female	36 (48.0%)	51 (45.1%)
Intact male	1 (1.3%)	5 (4.4%)
Castrated male	37 (49.3%)	56 (49.6%)
Age (years)		
Median [range]	10.5 [5.6–14.7]	11.2 [4.4–15.4]
Bodyweight (kg)		
Median [range]	18.1 [2.5–48.0]	19 [2.6–53.0]
Breed		
Mixed breed	13 (17.3%)	28 (24.8%)
Golden retriever	7 (9.3%)	10 (8.9%)
Boston terrier	5 (6.7%)	2 (1.8%)
Labrador retriever	5 (6.7%)	5 (4.4%)
Yorkshire terrier	4 (5.3%)	5 (4.4%)
Jack Russell terrier	3 (4.0%)	3 (2.7%)
Poodle (standard)	3 (4.0%)	
Siberian husky	3 (4.0%)	8 (7.1%)
Beagle	2 (2.7%)	8 (7.1%)
Border Collie	2 (2.7%)	3 (2.7%)
Shih Tzu	1 (1.3%)	4 (3.5%)
German Shorthaired Pointer		4 (3.5%)
Australian Shepherd	1 (1.3%)	3 (2.7%)
Other^a^	27 (36.0%)	22 (19.5%)

^a^ Other breeds include: Airedale terrier, American Eskimo, Alaskan malamute, beagle, Belgian malinois, bichon frise, Brittany spaniel, cairn terrier, cocker spaniel, coonhound, dachshund, English bulldog, English springer spaniel, feist terrier, Finnish spitz, flat‐coated retriever, German shepherd, Glen of Imaal terrier, Pembroke Welsh corgi, German shepherd, Italian greyhound, Lhasa apso, Maltese, miniature Australian shepherd, miniature poodle, miniature pinscher, mountain view cur, Norfolk terrier, Papillon, Pomeranian, pug, rat terrier, Rhodesian ridgeback, Samoyed, Schnauzer, Scottish terrier, shar‐pei, Shetland sheepdog, toy poodle, vizsla, Weimaraner, and West Highland white terrier.

### 3.1 Clinical findings at time of initial surgery

Preoperative haematology and serum biochemistry results were available for 69 cases (92.0%) and 111 controls (98.2%). All cases had hematologic abnormalities while two controls had normal bloodwork (Table 3). Most frequently noted abnormalities were elevations in liver enzymes. Preoperative thoracic imaging was performed in 85.3% (64/75) of cases and 91.2% (103/113) of controls. Pulmonary nodules or masses were noted in 4.0% (3/75) of cases and no controls. Abdominal imaging was performed in 98.7% (74/75) of cases and 99.1% (112/113) of controls. Hepatic tumours were most frequently noted in the left liver division (64.0% cases (48/75) and 56.6% controls (64/113)). Fine needle aspirate of the hepatic mass was performed in 66.7% (50/75) of cases and 65.5% (74/113) of controls. Fine needle aspirate was consistent with carcinoma, hepatocellular carcinoma, or hepatocellular neoplasia in 30.0% (15/50) of cases and 29.2% (21/74) of controls. Tumour dimensions were reported in 92.0% (69/75) of cases with median tumour diameter along the widest axis being 10 cm (range 0.5–24 cm). Tumour dimensions were reported in 90.2% (102/113) of controls with median tumour diameter along the widest axis being 10 cm (range 2.3–20 cm). Additional details of clinical findings can be found in Table 3.

**TABLE 3:** Clinical and diagnostic imaging findings in 75 dogs experiencing HCC tumour recurrence and 113 dogs not experiencing recurrence.

	Cases—Initial (*n* = 75)	Cases—At recurrence (*n* = 75)	Controls (*n* = 113)
Preoperative haematology Abnormalities	*n* = 69	*n* = 70	*n* = 111
Thrombocytosis	21 (30.4%)	27 (38.6%)	36 (32.4%)
Thrombocytopenia	3 (4.3%)	0	3 (2.7%)
Anaemia	27 (39.1%)	22 (31.4%)	28 (25.2%)
Neutrophilia	16 (23.2%)	11 (15.7%)	24 (21.6%)
Preoperative biochemistry abnormalities	*n* = 69	*n* = 70	*n* = 111
Elevated alanine aminotransferase (ALT)	60 (87.0%)	53 (75.7%)	93 (83.7%)
Elevated alkaline phosphatase (ALP)	61 (88.4%)	58 (82.9%)	95 (85.6%)
Elevated gamma‐glutamyl transferase (GGT)	19 (27.5%)	16 (22.9%)	33 (29.7%)
Elevated cholesterol	20 (29.0%)	22 (31.4%)	20 (18.0%)
Elevated blood urea nitrogen (BUN)	5 (7.2%)	10 (14.3%)	12 (10.8%)
Coagulation testing	*n* = 3	*n* = 0	*n* = 20
Prolonged PT/PTT	3 (100%)		9 (45%)
Preoperative Thoracic Imaging	*n* = 64	*n* = 56	*n* = 103
3 view thoracic radiographs	42 (65.6%)	37 (66.0%)	63 (61.2%)
Thoracic computed tomography (CT)	20 (31.3%)	16 (28.6%)	30 (29.1%)
Both radiographs and CT	2 (3.1%)	3 (5.4%)	10 (9.7%)
Evidence of pulmonary mass or nodules	3 (4.7%)	9 (16.1%)	0
Preoperative Abdominal Imaging	*n* = 74	*n* = 73	*n* = 112
3 view abdominal radiographs	1 (1.4%)	0	1 (0.9%)
Abdominal ultrasound	38 (51.4%)	47 (64.4%)	59 (52.7%)
Abdominal computed tomography (CT)	21 (28.4%)	15 (20.5%)	34 (30.4%)
Both ultrasound and CT	14 (18.9%)	11 (15.1%)	18 (16.1%)
Tumour location (lobe)			
Left lateral	27 (36.0%)	3 (4.0%)	33 (29.2%)
Left medial	12 (16.0%)	2 (2.7%)	29 (25.7%)
Caudate	6 (8.0%)	2 (2.7%)	12 (10.6%)
Quadrate	3 (4.0%)	2 (2.7%)	8 (7.1%)
Papillary process	0	0	3 (2.7%)
Right medial	6 (8.0%)	2 (2.7%)	12 (10.6%)
Right lateral	8 (10.7%)	4 (5.3%)	7 (6.2%)
Single mass involving multiple adjacent lobes^a^	12 (16.0%)	4 (5.3%)	8 (7.1%)
Not reported	1 (1.3%) (Right)	57 (76.0%)	1 (0.9%) (Left)
Tumour location (division)			
Left	48 (64.0%)	15 (20.0%)	64 (56.6%)
Central	13 (17.3%)	10 (13.3%)	20 (17.7%)
Right	14 (18.7%)	23 (30.6%)	23 (20.4%)
Left and central		1 (1.3%)	3 (2.7%)
Right and central		1 (1.3%)	1 (0.9%)
Not reported		26 (34.7%)	
Tumour dimensions along widest axis (cm)	*n* = 69	*n* = 23	*n* = 102
Median [range]	10 [0.5–24]	7.8 [3–18]	10 [2.3–20]
Hepatic mass fine needle aspirate findings	*n* = 50	*n* = 36	*n* = 74
Carcinoma, hepatocellular carcinoma, or hepatocellular neoplasia	15 (30.0%)	21 (58.3%)	21 (29.2%)
Marked atypia, likely consistent with hepatocellular neoplasia	13 (26.0%)	5 (13.9%)	22 (30.6%)
Mild atypia, vacuolar hepatopathy, or hyperplasia	9 (18.0%)	8 (22.2%)	20 (27.8%)
Inconclusive/nondiagnostic	13 (26.0%)	2 (5.6%)	9 (12.5%)

^a^ Single mass involving multiple lobes: initial findings for recurrent cases – left lateral and left medial (7); quadrate and right medial (3); quadrate, left lateral, and left medial (1); and quadrate and papillary process (1). At recurrence – one each caudate and right lateral, caudate and right medial, left medial and right lateral, and quadrate and left medial. Controls – caudate and left lateral (1); caudate and right lateral (1); left lateral and left medial (1); left lateral and right medial (1); left medial and papillary process (1); quadrate, caudate and right medial (1); quadrate and left medial (2).

### 3.2 Surgical complications at initial surgery

Nineteen cases (25.3%, 19/75) and 18 controls (15.9%, 18/113) experienced intraoperative complications. Complications are summarized in Table 4. The most common intraoperative complication was haemorrhage (18.7% of cases (14/75), 12.4% of controls (14/113)). Injury to adjacent soft tissue structures occurred in 5.3% (4/75) of cases and 2.7% (3/113) of controls and involved the caudal vena cava (4), diaphragm (2), and liver (1). Of patients experiencing complications, 52.6% (10/19) of cases and 50% (9/18) of controls required at least one transfusion of packed red blood cells, whole blood, or fresh frozen plasma.

**TABLE 4:** Perioperative complications in 75 dogs experiencing HCC tumour recurrence, 23 dogs undergoing treatment at the time of recurrence, and 113 dogs not experiencing recurrence.

	Cases—initial surgery (*n* = 75)	Cases—treatment at recurrence (*n* = 23)	Controls (*n* = 113)
Overall intraoperative complications			
Grade 0	55 (73.3%)	15 (65.2%)	95 (84.1%)
Grade 1	8 (10.7%)	6 (26.1%)	6 (5.3%)
Grade 2	9 (12.0%)	1 (4.3%)	9 (8.0%)
Grade 3	2 (2.7%)	1 (4.3%)	4 (3.5%)
Intraoperative haemorrhage	*n* = 14	*n* = 6	*n* = 14
Grade 1	5 (35.7%)	4 (66.7%)	6 (42.9%)
Grade 2	7 (50.0%)	2 (33.3%)	7 (50.0%)
Grade 3	2 (14.3%)	0	1 (7.1%)
Patients requiring at least one intraoperative transfusion	10 (13.3%)	2 (8.7%)	9 (8.0%)
Intraoperative Hypotension	*n* = 4	*n* = 0	*n* = 3
Grade 1	1 (25.0%)		0
Grade 2	3 (75.0%)		3 (100%)
Grade 3	0		0
Intraoperative Injury to adjacent soft tissue structures^a^	*n* = 4	*n* = 2	*n* = 3
Grade 1	0	0	0
Grade 2	2 (50.0%)	0	1 (33.3%)
Grade 3	2 (50.0%)	2 (100%)	2 (66.7%)
Overall Postoperative Complications			
Grade 0	61 (81.3%)	20 (87.0%)	97 (85.8%)
Grade 1	2 (2.7%)	0	7 (6.2%)
Grade 2	10 (13.3%)	2 (8.7%)	7 (6.2%)
Grade 3	1 (1.3%)	1 (4.3%)	2 (1.8%)
Postoperative Haemorrhage	*n* = 10	*n* = 0	*n* = 5
Grade 1	1 (10.0%)		0
Grade 2	8 (80.0%)		4 (80.0%)
Grade 3	1 (10.0%)		1 (20.0%)
Patients requiring at least one post‐operative transfusion	9 (12.0%)	1 (4.3%)	5 (4.4%)
Postoperative hypotension	*n* = 2	*n* = 0	*n* = 0
Grade 1	1 (50.0%)		
Grade 2	1 (50.0%)		
Grade 3	0		
Other complications			
Aspiration pneumonia (Grade 1)			1
Pneumothorax (Grade 1)			1
Surgical site infection (Grade 1)	1		1
Cardiac arrest—return of spontaneous function with CPR			1
Inoperable mass		2	
Congestive heart failure (Grade 3)		1	
Pancreatitis (Grade 2)		1	
Reoperation required	1 (1.3%)		1 (0.9%)

^a^ Injury to adjacent soft tissue structures included the caudal vena cava (4), diaphragm (2), hepatic vein (1), gallbladder (1) and adjacent liver (1).

Thirteen cases (17.3%, 13/75) and sixteen controls (14.2%, 16/113) experienced postoperative complications. The most common postoperative complication was haemorrhage (77.0% cases (10/13), 31.3% controls (5/16)). Of patients experiencing postoperative complications, 60.0% of cases (9/15) and 31.3% of controls (5/16) required at least one transfusion of packed red blood cells, whole blood, or fresh frozen plasma. One case and one control required reoperation due to persistent hemoabdomen. Liver division, size of the mass, and patient body weight were not significantly associated with the occurrence of perioperative complications.

### 3.3 Histologic findings from initial surgery

Histologic surgical margin results were reported in 49.3% (37/75) of cases and 27.4% (31/113) of controls. Margins were complete in 51.4% (19/37) of cases and 64.5% (20/31) of controls with margin results reported. The median histologic margin was 1.5 mm (range 1–150 mm) for cases and 5.0 mm (range 0.1–25 mm) for controls. Details of histologic margins, overall differentiation, nuclear pleomorphism, amount of necrosis, and mitotic count are summarized in Table 5.

**TABLE 5:** Histologic findings at initial surgery in 75 dogs experiencing HCC recurrence, 20 dogs undergoing surgery at time of HCC recurrence, and 113 dogs not experiencing HCC recurrence.

	Cases (*n* = 75)	Surgery at recurrence (*n* = 20)	Controls (*n* = 113)
Margins (mm)	*n* = 37	*n* = 12	*n* = 31
Complete	19 (51.4%)	6 (50.0%)	20 (64.5%)
Median [Range]	1.5 [1–150]	2 [2–30]	5 [0.1–25]
Degree of differentiation	*n* = 54	*n* = 18	*n* = 86
Well differentiated	47 (87.0%)	15 (83.3%)	76 (88.3%)
Moderately differentiated	6 (11.1%)	2 (11.1%)	7 (8.1%)
Poorly differentiated	1 (1.9%)	1 (5.6%)	3 (3.5%)
Nuclear pleomorphism	*n* = 56	*n* = 14	*n* = 73
Mild	31 (55.4%)	7 (50.0%)	43 (58.9%)
Moderate	22 (39.3%)	6 (42.9%)	26 (35.6%)
Marked	3 (5.4%)	1 (7.1%)	4 (5.5%)
Amount of necrosis	*n* = 59	*n* = 13	*n* = 57
None	10 (16.9%)	2 (15.4%)	2 (3.5%)
Mild	14 (23.7%)	4 (30.8%)	14 (24.6%)
Moderate	20 (33.9%)	5 (38.5%)	17 (29.8%)
Marked	25 (42.4%)	2 (15.4%)	25 (42.1%)
Mitotic count	*n* = 56	*n* = 19	*n* = 75
Median [range]	2 [0–37]	4 [0–23]	2 [0–22]

### 3.4 Tumour recurrence

Local tumour recurrence was documented at a median of 367 days (range 32–2096 days). Tumour recurrence was found during a routine restaging appointment (40.0%, 30/75), at a follow‐up appointment due to clinical signs including vomiting and inappetence (29.3%, 22/75) or elevated liver values (25.3%, 19/75), or during a workup for another disease process (5.3%, 4/75). For patients where recurrence was found at routine restaging, the median time to recurrence was 228 days (range 32–1099 days). Thoracic imaging was repeated at recurrence in 74.6% (56/75) of cases with suspicion of pulmonary metastasis noted in 16.1% (9/56. Tumour recurrence was suspected on abdominal ultrasound (64.4%, 47/75), CT (20.5%, 15/75), or a combination of abdominal ultrasound and CT (15.1%, 11/75). Fine needle aspirate cytology of the recurrent hepatic mass was performed in 48.0% (36/75) of cases. The detailed clinical findings at tumour recurrence are provided in Table 3. Tumour dimensions were reported in 24 cases with median recurrent tumour diameter along the widest axis being 7.8 cm (range 3–18 cm).

Twenty‐three cases underwent additional treatment to address their local tumour recurrence. Twenty dogs had surgery, one dog had microwave ablation and two dogs received chemoembolization. Fifty‐two cases elected for non‐surgical management including medical management (78.8%, 41/52), no further intervention (15.4%, 8/52), or euthanasia (5.8%, 3/52). Medical management consisted of chemotherapy agents, liver supportive medications, gastrointestinal supportive medications, and analgesics including non‐steroidal anti‐inflammatories. See Table 6 for additional details.

**TABLE 6:** Reason for re‐evaluation, method of identification of recurrence, treatment modality at recurrence, and time to recurrence in 75 dogs with recurrent HCC

	Recurrent cases (*n* = 75)
Reason for re‐evaluation	
Routine restaging	30 (40.0%)
Clinical signs (vomiting, inappetence, abdominal distension)	22 (29.3%)
Elevated liver values	19 (25.3%)
Work up for another disease	4 (5.3%)
Method of identification of recurrence	
Abdominal ultrasound	47 (62.7%)
Abdominal computed tomography (CT)	15 (20.0%)
Both ultrasound and CT	11 (14.7%)
Treatment modality at recurrence	
Surgical resection	20 (26.7%)
Microwave ablation	1 (1.3%)
Chemoembolization	2 (2.7%)
Medical management	41 (54.7%)
Chemotherapy^a^	9 (21.9%)
Liver support medications	8 (19.5%)
Gastrointestinal support medications	7 (17.1%)
Analgesia including non‐steroidal anti inflammatory	2 (4.9%)
No further treatment	8 (10.7%)
Euthanasia	3 (4.0%)
Time from Initial Surgery to Recurrence in All Dogs (days)	
Median [range]	367 [32–2096]
Time from Initial Surgery to Recurrence in Dogs with Routine Restaging (days)	*n* = 30
Median [range]	228 [32–1099]

^a^ Chemotherapy consisted of carboplatin (2), chlorambucil (1), Toceranib (6), cyclophosphamide (2), doxorubicin (1), gemcitabine (1), dasatinib (1), and vinorelbine (1) with 5 patients receiving multiagent therapy.

Sex, breed, age, body weight, tumour size, tumour location, FNA cytology findings, histologic findings, mitotic count, and completeness of excision were evaluated as risk factors for HCC recurrence. None were found to be significant risk factors in univariable conditional logistic regression.

### 3.5 Additional intervention for tumour recurrence

Of the 23 cases that underwent additional intervention for the treatment of local recurrence, 34.8% (8/23) experienced intraoperative complications (Table 4). The most common complication was haemorrhage (60.0%, 6/10). Despite this, none of the dogs undergoing additional surgical intervention required transfusion. One dog underwent chemoembolization intraoperatively due to the hepatic mass being deemed inoperable. This dog became hypotensive with grade 2 haemorrhage requiring a blood transfusion. The dog that underwent microwave ablation sustained grade 2 haemorrhage due to hepatic vein puncture and required a blood transfusion. One other mass was considered inoperable due to the proximity of the tumour to the caudal vena cava, and the surgery was aborted. One dog had substantial adhesions of the liver mass to surrounding structures necessitating a cholecystectomy in addition to the liver lobectomy. Histologic surgical margins were reported in 60.0% (12/20) of cases that underwent surgery for treatment of local tumour recurrence and were classified as complete in 50.0% (6/12). The median histologic margin distance was 2 mm (range 2–30 mm). Further histologic information is detailed in Table 5.

### 3.6 Outcomes

**FIGURE 1:**
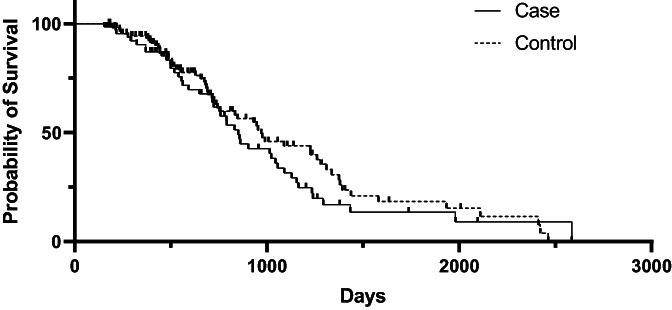
Kaplan–Meier survival curves for overall survival time for dogs with HCC who developed recurrence (case) or did not (control).

**FIGURE 2:**
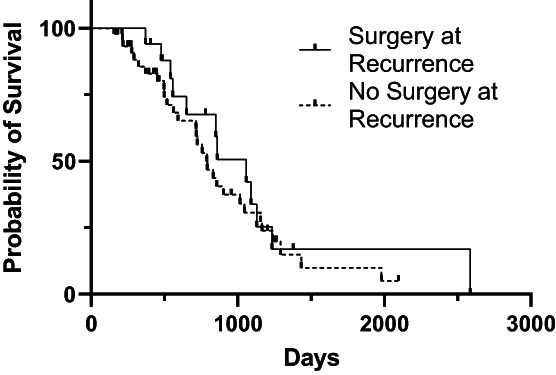
Kaplan–Meier survival curves for overall survival time for dogs with HCC who did or did not have surgery at the time of recurrence.

**FIGURE 3:**
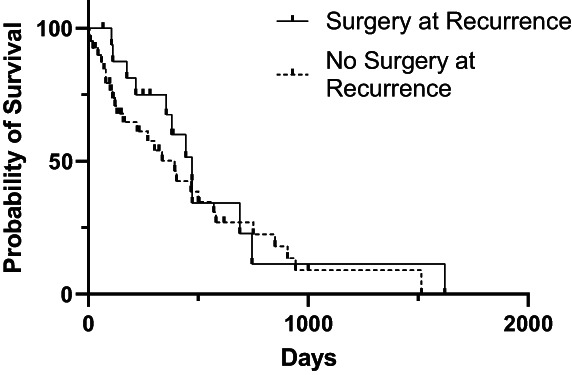
Kaplan–Meier survival curves for post recurrence survival time for dogs with HCC who did or did not have surgery at the time of recurrence.

**FIGURE 4:**
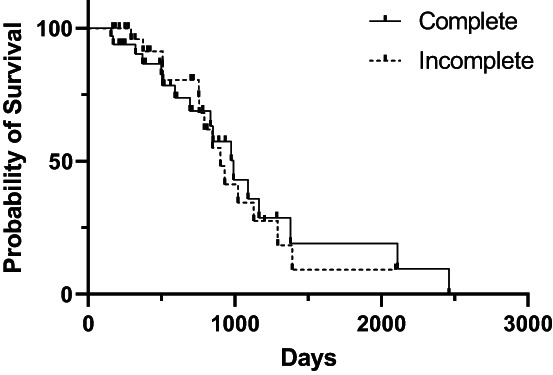
Kaplan–Meier survival curves for overall survival time for dogs with HCC who had complete or incomplete resection at initial surgery.

**FIGURE 5:**
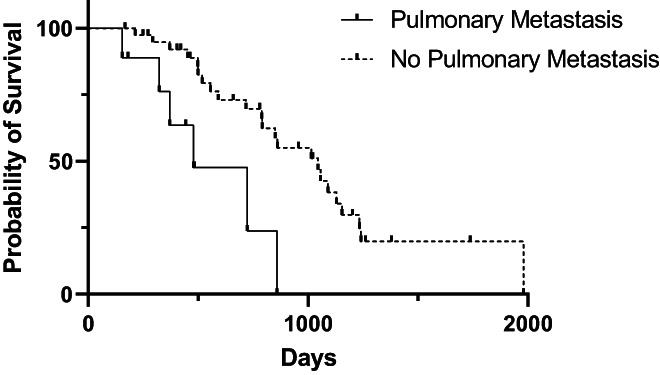
Kaplan–Meier survival curves for overall survival time for dogs with HCC who had suspect pulmonary metastasis on thoracic imaging at the time of HCC recurrence or did not.

At the time of case accrual, 22.7% (17/75) cases and 46.9% (53/113) controls were alive. The date of death was available for 44 cases and 57 controls. The median OS from the date of first surgery was 851 days [95% CI: 717, 1045]) for cases and 970 days [95% CI: 743, 1260] for controls (Figure 1). For cases, median OS for those that underwent additional treatment at tumour recurrence and those that did not was not significantly different (1057 days vs. 789 days; *p* = 0.45) (Figure 2). Median post recurrence OS was not significantly different for cases that had surgery at recurrence (472 days [95% CI: 159, 570]) and those that did not have surgery at recurrence (393 days [95% CI: 159, 570]; *p* = 0.45) (Figure 3). Median OS was not significantly different for dogs with histologically complete vs. incomplete margins (990 days [95% CI: 694, 1381] vs. 903 days [95% CI: 754, 1293]; *p* = 0.84) (Figure 4). When a cause of death was listed or necropsy findings were available, 70.4% (19/27) of cases died of causes most likely related to their recurrent HCC. Cause of death was reported as euthanasia due to progressive local disease or recurrence (57.9%, 11/19), euthanasia for hemoabdomen (26.3%, 5/19), euthanasia for marked abdominal effusion (transudate) with progressive liver disease (10.5%, 2/19), and death secondary to severe pancreatitis and pneumonia following surgery to address recurrence (5.2%, 1/19). As no controls were documented to have died due to causes most likely related to HCC, the disease specific median survival for controls could not be calculated. Overall survival for the nine cases with suspicion for pulmonary metastasis at the time of recurrence was 478 days [95% CI: 155, could not be estimated] which was significantly shorter than those without pulmonary metastasis (1045 days [95% CI: 789, 1155]) at the time of recurrence (*p* = 0.002) (Figure 5). For patients alive at the time of case accrual, the median time of follow‐up was 659 days (range 167–1739 days) for cases and 476 days (range 179–2009 days) for controls. Further outcome information is provided in Table 7.

## 4 DISCUSSION

The present study provides information on the largest case series to date of dogs with massive HCC treated surgically, with a large subset of cases documented to have a local recurrence of HCC. In this study population, the median time to develop tumour recurrence was 367 days (range 32–2096 days). Median time to recurrence was shorter in the 40.0% of cases undergoing routine restaging (228 days, range 32–1099 days). None of the risk factors evaluated in this study appeared to be significant for the development of recurrence. In comparing dogs that developed recurrence of HCC (cases) with those that did not (controls) there was no significant difference in the median OS (851 days for cases, 970 days for controls). For cases that underwent additional treatment at the time of recurrence, survival trended toward being longer but was not significantly different from those that did not undergo additional treatment (1057 vs. 789 days). There was no significant difference in median OS for dogs with histologically complete or incomplete margins (990 vs. 903 days). Dog with suspect pulmonary metastasis at the time of recurrence had a significantly shorter survival then dogs without pulmonary metastasis (478 vs. 1045 days).

**TABLE 7:** Outcome of 75 dogs experiencing HCC recurrence and 113 dogs without recurrence.

	Recurrent Cases (*n* = 75)	Controls (*n* = 113)
Patient status at time of accrual		
Alive	17 (22.7%)	53 (46.9%)
Dead	44 (58.7%)	57 (50.4%)
Lost to follow up	14 (18.7%)	3 (2.7%)
OS^a^ (days) Median [95% CI]	851 [717, 1045]	970 [743, 1260]
PFI^b^ (days) Median [Range]	367 [32–2096]	
Time to last follow up^c^ (days)	*n* = 15	*n* = 53
Median [range]	659 [249–1738]	476 [179–2009]
OS (days) pulmonary metastasis at recurrence	478 [155, could not be estimated]	
Median [95% CI]		
Survival %		
1 year	90.4%	94.4%
2 year	61.8%	64.4%
3 year	31.5%	44.0%
5 year	13.6%	18.4%

^a^ Overall survival time (OS)—time from initial surgery to date of death (when provided).

^b^ Progression free interval (PFI) = time from initial surgery to recurrence.

^c^ Time to last follow up—time from surgery to last follow up for cases alive at time of accrual.

In our study, the median time to local recurrence for dogs with HCC was around 1 year. Although previous studies have reported incidence of recurrence ranging from 0% to 27%^1^, ^7^ in dogs, none have specifically reported the timeframe to recurrence in a population of this size. Risk factors including sex, breed, age, body weight, tumour size, tumour location, FNA cytology findings, histologic findings, mitotic count, and completeness of excision were evaluated. None were found to be significantly associated with HCC recurrence in our population. Similarly, excluding age, Welsh corgi, and beagle breeds, none of these factors have previously been associated with the development of HCC.^5^ In humans, HCC accounts for approximately 80% of liver tumours with a 5 years postoperative recurrence rate of 70%.^11^, ^12^ One study including 661 people with HCC found that the majority of recurrences develop within the first year following resection, with a median time to recurrence of 22 months.^12^

Although our study did not identify any specific risk factors for recurrence, we did identify clinical features which may be useful in surveillance. Elevations in liver values were noted in dogs with recurrent disease with elevations in ALT and ALP in 74.6% and 83.1% of cases at recurrence, respectively. Bloodwork was evaluated only at the time of initial work up and during work up for recurrence. Due to this, it is unknown if a patient's bloodwork completely normalized during the intervening timeframe. However, as elevated liver enzymes are often what prompts initial work up and were the reason for reevaluation in 25% of patients with recurrent disease, it is reasonable to assume that those values were increased beyond that patients normal prompting restaging. Thus, liver enzyme elevations beyond a dogs post‐operative normal may represent a non‐invasive monitoring modality and should elicit further evaluation in patients with a history of HCC. Abdominal ultrasound was used to identify recurrence in 62.7% of cases and represents a relatively accessible and minimally invasive modality for disease monitoring. In humans with HCC, routine surveillance consisting of ultrasound and biomarker testing is performed every 3–6 months depending on the level of patient risk with the frequency adjusted based on individual patient factors.^13^ In our study, routine restaging was only performed in 40.0% of cases and did not have set timeframes or modalities for surveillance. Contributing authors reported recommended restaging intervals varying from no recommended routine restaging to every 3–6 months. The median time to identification of recurrence was shorter (228 days) in dogs who had routine restaging. Since information regarding the timing of every restaging event for each patient was not available, it is possible that the overall time to recurrence of 367 days may be an overestimation. This highlights the importance of developing individualized surveillance plans and continuing restaging beyond 1 year to catch recurrence at a point where treatment is feasible if the owner would be so inclined.

Biomarkers such as alpha‐fetoprotein (AFP), cell‐free DNA (cfDNA), and circulating tumour cells (CTC) are used in humans to help predict and identify HCC recurrence.^12^ Circulating tumour cell presence represents an intermediate stage between localized disease and distant metastasis. Changes in CTC before and after treatment can be used to accurately predict HCC recurrence in humans. Circulating tumour cell analysis has been explored in other solid tumours in dogs,^14^, ^15^ but has not been evaluated in the prediction of HCC recurrence in dogs. Due to the generally quiet biologic behaviour and overall low‐metastatic rate seen in canine HCC, CTC may be of limited utility but warrants further exploration. Cell‐free DNA is released into circulation from either dead or viable cancer cells and has been shown to identify human patients with disease recurrence or progression before changes can be detected on imaging.^12^ A recent study exploring quantification of plasma cfDNA levels in dogs with various tumours, including three hepatic tumours, found that plasma cfDNA concentrations were significantly elevated in dogs with malignant tumours.^16^ Both of these biomarkers have the potential for use in dogs to identify early recurrence as well as those at increased risk for recurrence who warrant a more stringent surveillance plan.

Our study found that survival for dogs undergoing treatment at the time of recurrence trended to be longer, although was not significantly different from dogs not undergoing treatment at recurrence. Treatment at recurrence may improve survival due to cytoreduction of the recurrent tumour. The lack of significance in our study population may be due to the small number of cases who had surgery at recurrence (26.3%, 20/76) or the slowly progressive nature of canine HCC. Surgeons may express reluctance to perform additional liver resections due to concerns for life‐threatening surgical complications secondary to tumour location or adhesions to surrounding structures. Although the overall complication rate was higher for surgery at recurrence (34.8% vs. 25.3% at initial surgery), 12.5% of those complications were grade 2 as opposed to 47.4% of complications being grade 2 at initial surgery. In the 20 dogs undergoing surgery at recurrence, none required a transfusion, one had adhesions to the gallbladder necessitating cholecystectomy, and only two liver masses were considered inoperable. No grade 3 complications occurred in cases treated surgically at recurrence, suggesting that with careful case selection surgical resection at recurrence may provide a survival benefit and be lower risk than previously believed. In humans with HCC recurrence, patients who undergo repeat treatment have been shown to have improved overall survival.^12^, ^17^ Treatment modalities utilized for human patients include repeat surgical resection, liver transplantation, ablation, transarterial chemoembolization (TACE), radioembolization, and systemic chemotherapy with curative intent surgical resection providing the greatest survival benefit.^24^

For dogs in which surgical resection of a liver tumour or local recurrence is not an option, other treatment modalities such as chemoembolization or microwave ablation can be utilized. The use of these modalities is reported in dogs with promising results.^18^, ^19^, ^20^, ^21^ In our study, assessment of the effect of chemotherapy on outcome was not possible due to the limited number of cases and the use of a variety of agents. A pilot study was recently published evaluating the use of sorafenib in dogs with unresectable nodular and diffuse HCC.^22^ Compared to metronomic chemotherapy with thalidomide, piroxicam, and cyclophosphamide, dogs treated with sorafenib had a significantly longer median time to progression and median overall survival time. Currently, there is no standard chemotherapy protocol and no widely proven survival benefit of chemotherapy for canine HCC.

As massive HCC is a slowly progressive disease in dogs with prolonged survival reported following complete or incomplete resection, it is not surprising that the case and control population did not differ significantly in OS. Our OS of 970 days for controls and 851 days for cases fits with survival times reported in other studies.^1^, ^7^, ^8^ The median OS trended to be longer for controls. This may be due to recurrent tumours being more aggressive in nature or lack of owner willingness to pursue additional treatment for recurrent cases. In humans, the development of recurrence is a poor prognostic indicator as these tumours tend to have a more aggressive biologic behaviour.^12^ However, human HCC is overall a more aggressive disease than canine HCC, with a high‐local recurrence and distant metastatic rate.^12^

Our study found that there was no significant difference in median OS for dogs who had histologically complete versus incomplete resection of their tumours (990 vs. 903 days). Conflicting reports exist regarding the survival benefit of complete excision. Previous studies have suggested a prolonged survival with complete excision compared to incomplete excision, but more recent literature does not support this.^1^, ^7^, ^23^ A recent retrospective study including 94 dogs found no significant difference in overall median survival time between completely and incompletely resected HCC.^8^ Margin reporting is variable between pathologists and was only reported in 49.3% of cases and 27.4% of controls in our study. Inconsistent or completely absent margin reporting may be due to the inability of the pathologist to determine the surgical margin, histopathology only evaluating a small segment of margin, or perceived unimportance of margin diameter which may lead to confounding in retrospective studies.

Pulmonary masses or nodules suspicious for HCC metastasis were noted on thoracic imaging at the time of recurrence in 16.1% (9/56) of cases. This is in accordance with the previously reported distant metastatic rate of 0%–37% for HCC^2^. A significant difference in overall survival was noted between cases with and without suspicion of pulmonary metastasis at the time of recurrence (478 vs. 1045 days). However, with only nine patients having evidence of pulmonary metastasis and only six of those having a date of death reported, it is difficult to draw any overarching conclusions from this finding as it may represent a type I error.

The retrospective and descriptive nature of this study carries inherent limitations. The histopathology slides not being read by a single pathologist may have created bias due to inconsistent reporting as no standard reporting system exists for canine liver tumours. As we found that recurrence happens around 1 year following surgery, the minimum six‐month follow‐up for controls could have resulted in the inclusion of some controls that may have later developed recurrence. As recurrence was noted with ultrasound in the majority of patients, and ultrasound has been found to correctly localize liver masses in only 51.8%–74% of cases, the inability of imaging reports to give specific locations of recurrence is not unusual but could have allowed inclusion of some cases who had new masses instead of recurrence.^24^, ^25^ Additionally, the lack of standardized routine restaging may have led to some overestimation of the time to recurrence.

In conclusion, surgical intervention remains the mainstay of treatment for dogs with massive HCC. Local recurrence occurred a median of 1 year from the time of initial surgery, although was noted earlier in dogs undergoing routine restaging. Overall survival time was not significantly different in dogs with or without recurrence. In dogs with recurrent HCC, treatment at recurrence trended toward prolonged OS but was not significantly different than for dogs not undergoing treatment at recurrence. Completeness of excision did not significantly impact OS. Dogs with suspicion of pulmonary metastasis at the time of recurrence had a significantly decreased overall survival compared to those without. However, this may represent a type I error and their median overall survival was still greater than 1 year. No risk factors were identified for the development of recurrence. A routine surveillance plan should be patient‐specific and extend beyond 1 year after the initial surgery. Canine patients with massive HCC have a good long‐term prognosis, even in the face of incomplete resection, pulmonary metastasis, and recurrent disease.

## CONFLICT OF INTEREST

The authors declare no conflict of interest.

## Data Availability

The data that support the findings of this study are available from the corresponding author upon reasonable request.
